# Characterisation and impact of intratumoural stroma in melanoma and carcinoma brain metastases

**DOI:** 10.1002/2056-4538.70061

**Published:** 2025-11-29

**Authors:** Dave Bandke, Serge Weis, Andreas Gruber, Petar Noack, Ognian Kalev, Karoline Ornig, Rupert Langer

**Affiliations:** ^1^ Institute of Clinical Pathology and Molecular Pathology Kepler University Hospital, Johannes Kepler University Linz Austria; ^2^ Clinical Research Institute for Neurosciences Johannes Kepler University Linz Austria; ^3^ Department of Neurosurgery Kepler University Hospital, Johannes Kepler University Linz Austria

**Keywords:** brain metastasis, tumour microenvironment, intratumoural stroma, desmoplasia, histomorphology, survival analysis

## Abstract

Research on the tumour microenvironment in brain metastases (BM) has predominantly focused on the immune response, while the presence, morphological patterns, and potential clinical relevance of intratumoural stroma remain less intensively investigated. We retrospectively analysed 604 BM (529 carcinomas, 75 melanomas) from 556 patients. Intratumoural stroma was histomorphologically classified into absent/unclear (Group 0), present without desmoplasia (Group 1) or with desmoplasia (Group 2). Associations with histological features, clinical parameters, and survival were evaluated. Intratumoural stroma was absent in 63.2% of tumours (*n* = 382), was present without desmoplasia in 14.9% (*n* = 90), and was desmoplastic in 21.9% (*n* = 132). Desmoplasia was most frequent in metastases from breast carcinomas and pulmonary squamous cell carcinomas. No significant associations were found between stroma groups and age, sex, brain location, PD‐L1 expression, oncogenic mutations in lung carcinoma, or breast‐cancer molecular subtype. Independent predictors of poorer survival were increasing age (HR = 1.029, 95% CI: 1.018–1.039, *p* < 0.001), male sex (HR = 1.492, 95% CI: 1.204–1.849, *p* < 0.001), and infratentorial location (HR = 1.402, 95% CI: 1.125–1.748, *p* = 0.003). Stroma groups showed no independent prognostic value in the overall cohort. However, subgroup analysis of non‐small cell lung cancer revealed a U‐shaped relationship, with Group 1 stroma linked to better survival (*p* = 0.020). In summary, intratumoural stroma in BM is a carcinoma‐specific phenomenon, absent in melanoma. Patient outcomes, however, were primarily determined by demographic and anatomical factors rather than stromal morphology in the overall cohort but may have clinical relevance in particular tumour subgroups.

## Introduction

Brain metastases (BM) are associated with significant physical and psychological burdens due to their symptoms, which include persistent headaches, neurological deficits and organic psychosyndromes. Diagnosis often marks the beginning of palliative therapy, as median survival rates range between 5 and 13 months [1, 2]. By far the most common BM originate from lung carcinomas, followed by breast and melanoma.

Although research on the cellular compartments of the tumour microenvironment of BM has significantly advanced our understanding of the immune response and reactive changes in brain tissue, the role of the intratumoural stroma itself remains an important area for future investigation [3, 4, 5, 6, 7].

In the strict sense, tumour stroma refers to the desmoplastic connective tissue that is specifically formed or remodelled by the tumour. Most BM have only a narrow, blood‐vessel‐rich stroma inside the tumour. Interestingly, some carcinoma metastases have a broader desmoplastic stroma, which has not yet been systematically characterised. This finding is particularly unusual because normal brain tissue does not contain fibroblasts.

Unlike their counterparts in normal wound healing, activated fibroblasts within primary tumours do not undergo apoptosis. The chronic tissue damage that accompanies tumour development instead drives them into a state of irreversible activation [8]. This leads to an altered extracellular matrix, which can appear as a greyish background within the connective tissue on haematoxylin and eosin (HE) staining.

Some BM also exhibit broad fibrous stroma despite lacking desmoplastic features. Additionally, the term ‘stroma’ is sometimes also used for changes in the surrounding reactive brain tissue, leading to misconceptions in the interpretation of findings about tumour microenvironment. The aim of our study was to better elucidate this interesting phenomenon and to investigate the occurrence and clinical relevance of intratumoural stroma in BM based on histomorphological findings. Using a large patient cohort, we developed a three‐tiered classification system to better differentiate and characterise these stromal patterns: Group 0 without significant stroma detection; Group 1 with collagenous stroma; and Group 2 with broader, greyish stroma resembling desmoplasia in extracranial tumours, which was termed intratumoural desmoplastic stroma (IDS). The results of the histopathological analyses were then correlated with other pathological and clinical parameters, including patient outcome.

## Materials and methods

### Patients and tissue material

Paraffin‐embedded tissue blocks from 1,099 metastases of 967 patients who underwent neurosurgical resection or biopsy between 2010 and 2019, were collected from the archive of the Neuropathology Unit, Institute of Pathology and Molecular Pathology, Kepler University Hospital. After excluding spinal metastases (*n* = 108), we included BM from primary tumour sites with ≥20 cases and adequate tissue (most biopsies excluded), yielding 604 BM from 556 patients; 42 patients had at least one additional resection of a second BM.

Primary tumours comprised 309 lung carcinomas (51.2%), 92 breast carcinomas (15.2%), 75 melanomas (12.4%), 37 colon carcinomas (6.1%), 30 gastric/oesophageal adenocarcinomas (5.0%), 26 kidney carcinomas (4.3%), 19 prostate carcinomas (3.1%), and 16 bladder carcinomas (2.6%).

Median age was 62.1 years. Male‐to‐female distribution was 55%:45%. Metastatic sites were cerebrum (*n* = 433, 71.7%), cerebellum (*n* = 147, 24.3%), and dura (*n* = 24, 4.0%).

This study was conducted in accordance with the principles of the Declaration of Helsinki and approved by the Ethics Committee of Upper Austria under approval number EK 1221/2021.

### Evaluation of stroma quality and content

FFPE blocks were newly cut to ensure uniform high‐quality staining. Sections were systematically assessed for morphologically recognisable stromal content and categorised into three groups.

**Figure 1:**
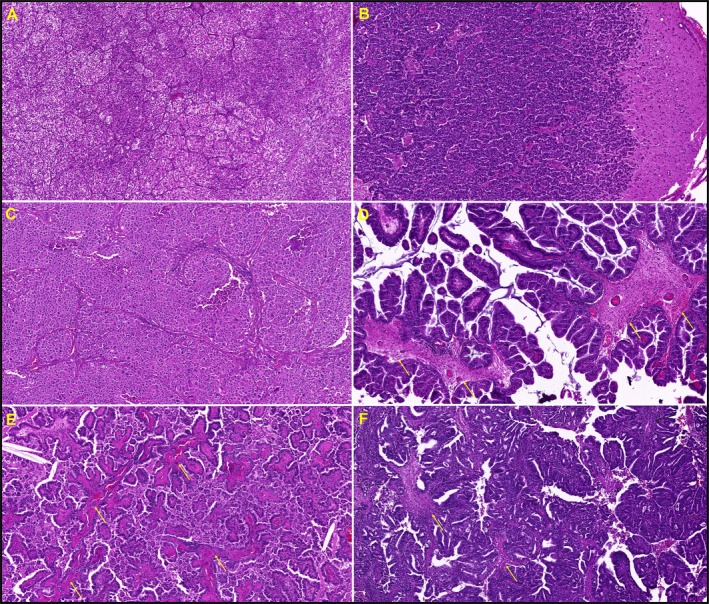
(A–C) Representative examples of three BM (×100 total magnification), assigned to stroma Group 0. These cases show only narrow perivascular connective tissue without the formation of an intratumoural stroma. (A) Clear cell renal cell carcinoma, typically forming small solid nests separated by a delicate capillary‐rich stroma. (B) Breast adenocarcinoma with diffuse infiltration of small tumour cells into the adjacent temporal lobe parenchyma without any intratumoural stroma. (C) Urothelial carcinoma with a predominantly solid growth pattern. (D–F) Representative examples of three BM (×100 magnification) exhibiting a recognisable fibrous stroma (highlighted by arrows) that, while clearly developed, does not appear desmoplastic. This group 1 stroma is typically associated with papillary growth patterns. (D and E) Lung adenocarcinomas. (F) Gastric adenocarcinoma.

Group 0 typically displayed solid cell nests or discohesive cell infiltrates separated by narrow capillaries with no clearly identifiable connective tissue apart from endothelial cells and pericytes. This pattern is not considered classical tumour‐associated stroma (Figure 1).

Group 1 exhibited expanded fibrous stroma, often with a papillary growth pattern (Figure 1).

**Figure 2:**
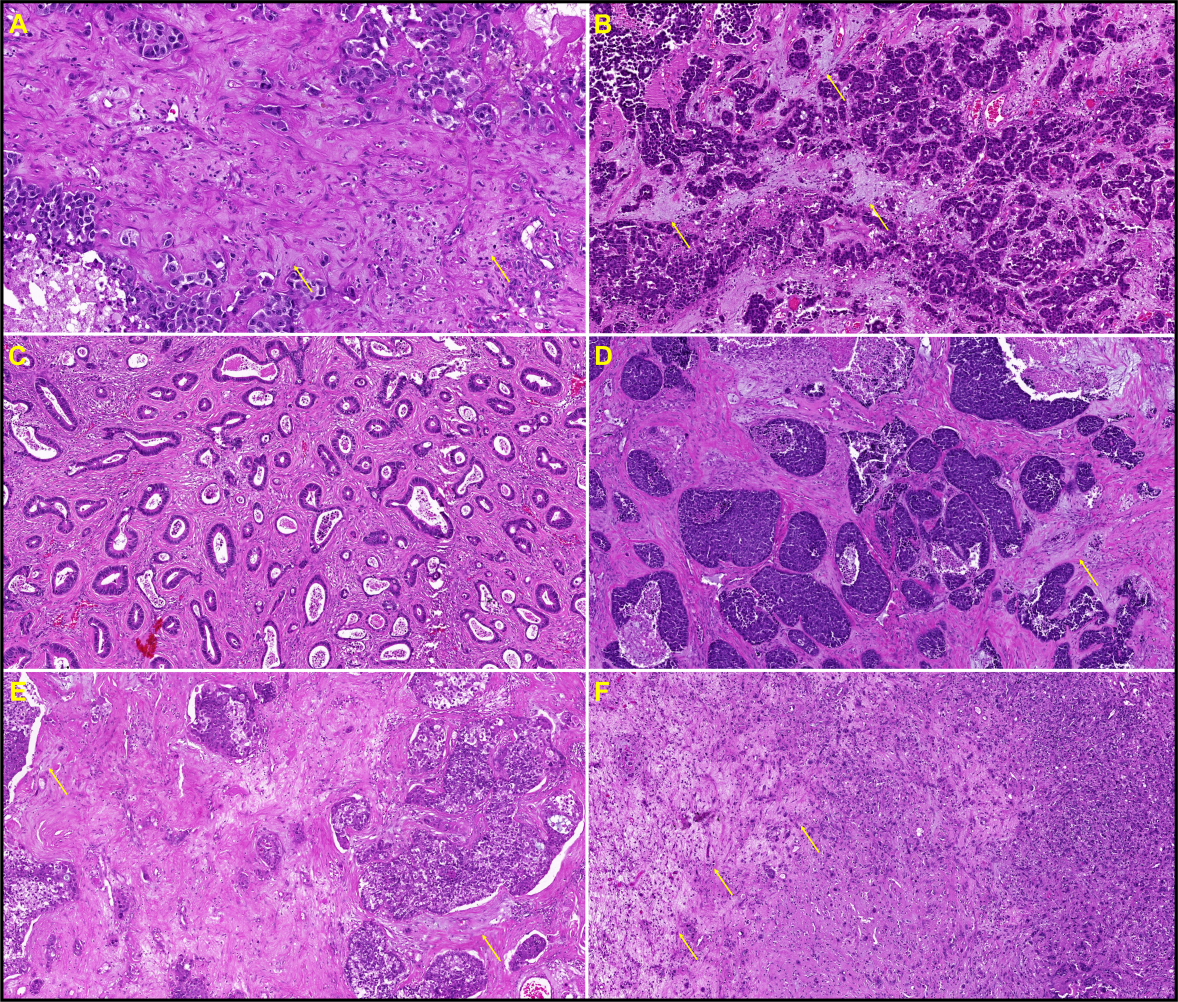
Representative examples of six BM (×100 total magnification) exhibiting intratumoural desmoplastic stroma (IDS), corresponding to stroma Group 2. Typically grey‐appearing areas in the broad stroma are highlighted by arrows. (A and B) Breast carcinomas. (C) Colorectal adenocarcinoma with tubular growth pattern. (D) Pulmonary squamous cell carcinomas. (E and F) Pulmonary adenocarcinomas.

Group 2 (=IDS) demonstrated at least focally broader, greyish stroma on HE, which in other organs or primary tumours corresponds to desmoplastic stroma, suggesting altered extracellular matrix (Figure 2).

### Statistical analyses

Analyses were performed in SPSS v22.0 (IBM). Categorical variables (sex, location, tumour types, molecular data) were compared using chi‐square tests. When several categories were evaluated simultaneously, Pearson's chi‐square was applied and association strength quantified by Cramer's V. Two‐sided *p* < 0.05 was considered significant. For multivariable analyses, Bonferroni correction was applied. Inter‐observer agreement for histomorphological classification was assessed and reliability quantified using Cohen's kappa.

Overall survival (OS) was defined as the interval from surgical resection to death. For patients with multiple resections, only the first resection and corresponding first metastasis were analysed. Effects on OS were evaluated by univariate Kaplan–Meier analysis with log‐rank tests and by multivariable Cox proportional hazards regression to account for potential covariates.

## Results

### Inter‐rater reliability analysis

Two pathologists (DB, PN) independently assigned 46 randomly selected digital slides to stroma groups 0–2. Identical scores occurred in 43 cases (raw agreement 93.5%). Chance‐corrected agreement was high (Cohen's *κ* = 0.90, 95% CI 0.73–1.00; quadratic‐weighted *κ* = 0.954). All three discrepancies differed by a single category (Group 0 versus 1); none were opposite extremes (Group 0 versus 2). Per Landis–Koch, agreement was ‘almost perfect,’ supporting robustness for subsequent analyses [9].

### Morphological analysis

Overall, 382 metastases lacked classical stroma (Group 0), 90 showed expanded non‐desmoplastic stroma (Group 1), and 132 had intratumoural desmoplastic stroma (Group 2, IDS). IDS occurred only in carcinoma brain metastases (24.9%) and in none of the 75 melanoma metastases (Table 1).

**Table 1:** Distribution of all 604 BM according to the presence or absence of intratumoural stroma

		Intratumoural stroma			Sum
		Group 0 (=no)	Group 1	Group 2 (=IDS)	
Sex	Male	216 (65.0%)	53 (16.0%)	63 (19.0%)	332 (100%)
	Female	166 (61.0%)	37 (13.6%)	69 (25.4%)	272 (100%)
Year	2010–2014	192 (63.3%)	48 (15.6%)	68 (22.1%)	308 (100%)
	2015–2019	190 (64.2%)	42 (14.2%)	64 (21.6%)	296 (100%)
Location	Cerebrum	282 (65.1%)	62 (14.3%)	89 (20.6%)	433 (100%)
	Cerebellum	82 (55.8%)	28 (19.0%)	37 (25.2%)	147 (100%)
	Dura	18 (75.0%)	0 (0%)	6 (25.0%)	24 (100%)
Primary	Lung carcinoma	188 (60.8%)	46 (14.9%)	75 (24.3%)	309 (100%)
	Adenocarcinoma	152	44	54	250
	Squamous carcinoma	7	1	14	22
	Neuroendocrine carcinoma	27	5	1	33
	Breast carcinoma	55 (59.8%)	3 (3.3%)	34 (36.9%)	92 (100%)
	Melanoma	75 (100%)	0 (0%)	0 (0%)	75 (100%)
	Colorectal carcinoma	5 (13.5%)	27 (73.0%)	5 (13.5%)	37 (100%)
	Oesophagogastric adenocarcinoma	12 (40.0%)	11 (36.7%)	7 (23.3%)	30 (100%)
	Kidney carcinoma	23 (88.5%)	0 (0%)	3 (11.5%)	26 (100%)
	Prostate carcinoma	14 (73.7%)	2 (10.5%)	3 (15.8%)	19 (100%)
	Urothelial carcinoma	10 (62.5%)	1 (6.25%)	5 (31.25%)	16 (100%)
Entity	Adenocarcinoma	261 (57.5%)	87 (19.2%)	106 (23.3%)	454 (100%)
	Squamous carcinoma	7 (31.8%)	1 (4.5%)	14 (63.6%)	22 (100%)
	Neuroendocrine CA	27 (81.8%)	1 (3.0%)	5 (15.2%)	33 (100%)
	Melanoma	75 (100%)	0 (0%)	0 (0%)	75 (100%)
	Carcinoma (no subtype)	12 (60.0%)	1 (5.0%)	7 (35.0%)	20 (100%)
Sum	*n* = 604	382 (63.2%)	90 (14.9%)	132 (21.9%)	604 (100%)

In cases where intratumoural stroma (Group 1 + 2) was identified, the presence of at least focal desmoplastic stroma (IDS, Group 2) was further assessed. The data are stratified by sex, 5‐year span, brain location, primary tumour origin, and histological carcinoma subtype.

Melanoma metastases typically present with pleomorphic, sometimes spindle‐shaped tumour cells, accompanied by vascular stroma without the development of a classical tumour stroma (neither Group 1 nor Group 2), with more frequent haemorrhagic areas, and often a marked lymphocytic infiltrate.

Among carcinomas, Pearson's chi‐square test revealed a statistically significant and moderate to strong association between different primary tumour types and stroma groups (Table 2: *p* < 0.001; Cramér's *V* = 0.339).

**Table 2:** Standardised residuals (SR) from the Pearson chi‐square test, where all values within the range of ±1.96 are considered non‐significant

Primary tumour	Stroma Groups		
	Group 0 (SR)	Group 1 (SR)	Group 2 (SR)
Colon	**−3.6**	**+8.3**	−1.4
Stomach/Oesophagus	−1.3	**+2.6**	**−2.2**
Breast	+0.2	**−3.2**	**+2.3**
Kidney	**+2.0**	**−2.1**	−1.4
Lung	+0.6	−0.9	−0.2
Prostate	+0.9	−0.7	−0.8
Bladder	+0.2	−1.0	−1.0

Residuals indicating a significantly higher‐than‐expected frequency are highlighted in bold green, whereas those reflecting a significantly lower‐than‐expected frequency are marked in bold red.

Lung adenocarcinomas showed the greatest morphological heterogeneity (papillary, solid, acinar, spindle‐shaped, small‐cell features) among all adenocarcinomas. Overall stroma‐group distribution in lung metastases (*n* = 309) resembled other carcinoma metastases. Subgroup analysis however showed IDS was uncommon in neuroendocrine carcinomas (NEC; *n* = 33) and more frequent in squamous cell carcinomas (SqCC; *n* = 22), resulting in a significant, moderately strong association across lung subtypes (*p* < 0.001; Cramér's *V* = 0.264). SqCCs were significantly more likely to exhibit a typical stromal pattern (SR = + 3.8). Thus, SqCCs had the highest IDS prevalence; NECs had the lowest.

Breast carcinomas often formed broad desmoplastic areas, sometimes spanning several square millimetres without epithelial components. Among all carcinoma types, breast carcinoma most frequently exhibited IDS (*n* = 34/92, 37.0%, SR = +2.3) and showed a significant deficit of Group 1 stroma (SR = −3.2), indicating that when stroma was present it was typically desmoplastic.

Gastrointestinal carcinomas (stomach/oesophagus and colon) frequently had intratumoural stroma, highest in colorectal metastases (*n* = 32/37, 86.5%). Colon carcinoma was the only entity with a significantly lower‐than‐expected number of cases lacking stroma (Group 0: SR = −3.6). Oesophageal/gastric carcinomas also commonly presented with intratumoural stroma (*n* = 18/30, 60.0%), though less often than colorectal cases. Both sites often displayed papillary/tubular growth with narrow, well‐defined stromal compartments and fewer greyish desmoplastic areas. Colorectal metastases showed a major surplus of Group 1 (SR = +8.3), while upper GI tumours were also overrepresented in Group 1 (SR = +2.6). IDS was less common overall, with a modest deficit in colorectal tumours (Group 2: SR = −1.4) and a significant deficit in stomach/oesophageal primaries (Group 2: SR = −2.2).

Renal‐cell carcinomas (RCC) exhibited the opposite extreme, frequently lacking any discernible stroma (Group 0: SR = +2.0, Group 1: SR = −2.1) and only rarely showing IDS (*n* = 3/26; 11.5%). Clear‐cell RCCs comprised pale, clear tumour cell nests separated by thin capillary septa. Of the three IDS‐positive RCCs, two had known papillary RCC primaries; one had an unknown original histology.

Prostate and urothelial carcinomas approximated the overall distribution. Prostate carcinomas were typically solid with infrequent IDS (*n* = 3/19, 15.8%). Urothelial carcinomas displayed predominantly solid morphology.

Among 42 patients with ≥1 additional BM resection, the stroma group remained constant in 34 cases (24 always Group 0; 4 always Group 1; 6 always Group 2). Eight showed discordance (three lung, two breast, two upper GI, one bladder); in each, IDS was present in one specimen but absent in the other. One patient had three cerebral metastases resected over 3 years: no IDS in 2011; clear IDS (=Group 2) in 2012/2013 (supplementary material, Table S1).

### Comparison intratumoural stroma with clinical and pathological parameters

In general, no significant results were observed across all stroma groups with respect to the following parameters. For clarity, we report only *p* values comparing IDS (=Group 2) to non‐IDS cases (Groups 0 + 1): No significant associations were observed between IDS and patient sex (*p* = 0.061), age (*p* = 0.887), brain location (cerebrum, cerebellum, dura; *p* = 0.279), primary organ of the metastasis (*p* = 0.062) or year of resection (2010–2019, *p* = 0.864).

In lung metastases, IDS showed no association with mutations in *EGFR* (*n* = 95, *p* = 0.703), *KRAS* (*n* = 77, *p* = 0.476), and *BRAF* (*n* = 71, *p* = 0.581), or PD‐L1 Tumour Proportion Score (TPS: >1% and >50%; *n* = 49, *p* = 0.880).

In breast metastases, IDS was unrelated to molecular subtype (*n* = 86, *p* = 0.450), ER (*n* = 86, *p* = 0.816), PR (*n* = 86, *p* = 0.529), or HER2 (*n* = 89, *p* = 0.822).

### Survival analysis

Survival was available for 501 patients (Table 3), ranging from 1 day to 175.6 months (14.6 years). Median survival was 10.3 months (IQR: 4.1–26.6 months); mean age at first resection was 62.1 years.

**Table 3:** Univariate analysis of median overall survival

		Median OS in months [95% CI]	*p*
Sex	Male (*n* = 274)	7.5 [5.9–9.1]	0.000
	Female (*n* = 227)	16.4 [13.5–19.3]	
Age	<50 years (*n* = 62)	20.3 [13.8–26.8]	0.000
	50–65 years (*n* = 243)	11.9 [9.9–13.9]	
	>65 years (*n* = 196)	6.7 [5.1–8.4]	
Location	Cerebrum (*n* = 355)	12.0 [10.0–14.2]	0.006
	Cerebellum (*n* = 129)	6.9 [4.7–9.2]	
Stroma (IDS)	No IDS (Group 0 + 1, *n* = 396)	10.9 [9.3–12.5]	0.056
	IDS (Group 2, *n* = 105)	9.1 [7.2–11.0]	
Stroma (group)	Group 0 (*n* = 319)	10.4 [8.5–12.3]	0.126
	Group 1 (*n* = 77)	14.3 [7.8–20.7]	
	Group 2, IDS (*n* = 105)	9.1 [7.2–11.0]	
Primary organ	Lung (*n* = 263)	10.5 [8.1–12.8]	0.019
	Breast (*n* = 77)	15.0 [9.0–21.0]	
	Melanoma (*n* = 56)	7.4 [5.1–13.2]	
	Colon (*n* = 34)	9.2 [6.9–13.1]	
	Upper GI (*n* = 23)	10.1 [7.0–13.0]	
	Kidney (*n* = 20)	11.1 [1.8–20.4]	
	Prostate (*n* = 15)	6.4 [4.7–8.2]	
	Bladder (*n* = 13)	7.4 [0.0–15.0]	
Tumour (histology)	Adenocarcinoma (*n* = 381)	11.2 [9.2–13.3]	0.050
	Squamous cell CA (*n* = 20)	9.8 [7.8–11.7]	
	Neuroendocrine CA (*n* = 28)	8.7 [5.6–11.8]	
	Carcinoma, no subtype (*n* = 16)	5.1 [0.0–11.9]	
	Melanoma (*n* = 56)	7.4 [5.1–13.2]	
*EGFR* (lung)	Wildtype (*n* = 74)	14.3 [10.5–18.0]	0.764
	Mutation, activating (*n* = 10)	14.8 [0.0–36.4]	
*KRAS* (lung)	Wildtype (*n* = 50)	14.3 [11.5–17.0]	0.463
	Mutation (*n* = 17)	20.2 [3.9–36.6]	
*BRAF* (lung)	Wildtype (*n* = 56)	15.1 [11.9–18.3]	0.967
	Mutation (*n* = 5)	32.3 [0.0–66.9]	
Breast (molecular type)	Luminal A: HR+, Her2‐ (*n* = 17)	28.2 [3.0–53.4]	0.104
	Luminal B: HR+, Her2+ (*n* = 10)	12.1 [0.0–31.7]	
	Her2 positive (*n* = 17)	24.7 [13.0–36.5]	
	Triple‐negative (*n* = 23)	8.0 [5.7–10.4]	
Overall	All patients (*n* = 501)	10.3 [8.9–11.7]	

The stromal component showed no independent prognostic significance. In multivariable Cox regression, only male sex, increasing age, and infratentorial tumour location remained significant adverse prognostic factors.

**Figure 3:**
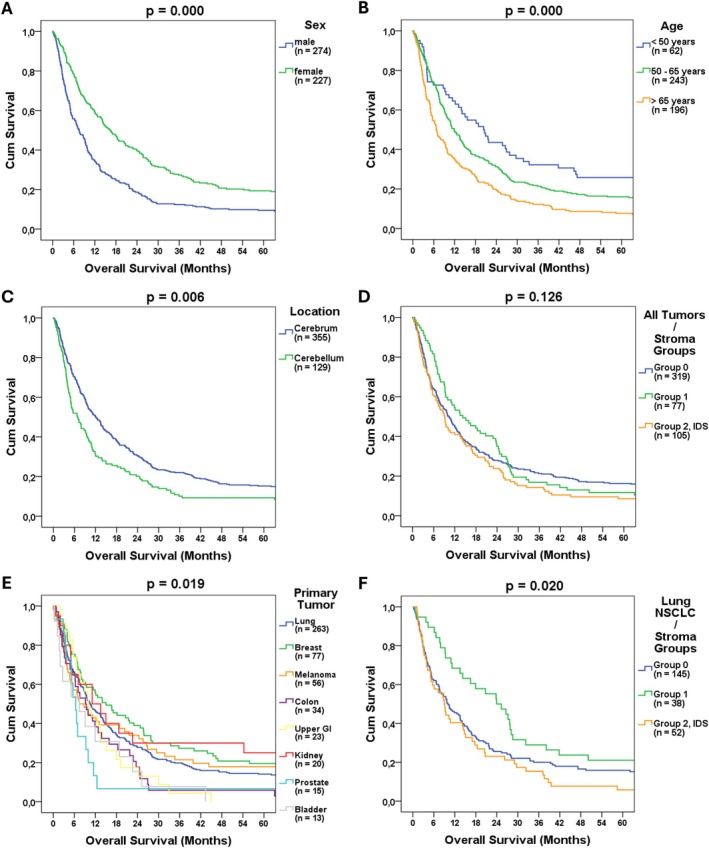
Kaplan–Meier survival curves of 501 patients, stratified by (A) sex, (B) age, (C) brain location, (D) stroma groups (overall), (E) primary organ of metastases, and (F) stroma groups within non‐small cell lung cancer (NSCLC). Overall survival in months was defined as the interval between the date of surgical resection and the date of death. Cum survival, cumulative survival.

No survival differences were observed among stroma Groups 0, 1, and 2 (Figure 3; *p* = 0.126). Stratification by IDS status showed a trend toward significance but did not reach statistical significance for survival differences (*p* = 0.056). In the univariate Kaplan–Meier analysis, the following factors were significantly associated with survival: age (*p* < 0.001), sex (*p* < 0.001), location of the BM (*p* = 0.006), primary organ of the metastasis (*p* = 0.019) and, with borderline significance, the histological subtype (*p* = 0.050). In a multivariable Cox proportional hazards regression (overall *p* < 0.001), only age, sex, and brain location remained significant. The diverging survival curves among tumour entities primarily reflect differences in the average age of the respective patient subgroups, for example, mean age for breast cancer was 57.2 years, and for prostate cancer 72.9 years.

Increasing age was significantly associated with reduced survival (HR = 1.029, 95% CI: 1.018–1.039, *p* < 0.001), corresponding to a 2.9% increase in the hazard of death per additional year. Male sex was also associated with higher mortality (HR = 1.492, 95% CI: 1.204–1.849, *p* < 0.001). Furthermore, patients with a metastasis in the cerebellum had a significantly higher hazard of death relative to cerebral metastasis (HR = 1.402, 95% CI: 1.125–1.748, *p* = 0.003).

### Subgroups lung cancer

Among lung cancers, SqCC and NEC appeared to fare worse than adenocarcinoma on visual assessment of the Kaplan–Meier curves, but survival did not differ significantly between histological subtypes (*n* = 260, *p* = 0.120). In a lung‐only multivariable Cox model, histological subtype (*p* = 0.288) or the presence of IDS (*p* = 0.067) was not significant; age (*p* = 0.002), male sex (*p* < 0.001), and infratentorial location (*p* = 0.034) were adverse.

However, in non‐small‐cell lung carcinomas (adenocarcinoma + SqCC), stroma groups were prognostic (Kaplan–Meier *p* = 0.020; Figure 3). In a multivariable Cox model including age, sex, brain location, and histology, stroma group remained independently predictive (*p* = 0.028). Compared with the reference stroma group (Group 1), tumours lacking typical intratumoural stroma (Group 0) showed a 51% increase in the odds of death (OR = 1.51, 95% CI: 1.05–2.17; *p* = 0.028), while those with intratumoural desmoplastic stroma (IDS) almost doubled the odds (OR = 1.83, 95% CI: 1.18–2.85; *p* = 0.007).

### Subgroups of breast cancer

Among breast carcinomas, the molecular subtypes of BM appeared to reflect the known prognostic differences observed in primary tumours based on visual assessment (Figure 3), with hormone receptor–positive tumours showing the most favourable outcomes, followed by HER2‐positive subtypes (with or without hormone receptor co‐expression), and triple‐negative tumours exhibiting the poorest outcomes.

However, in the univariate analysis, no statistically significant difference in survival was observed (*p* = 0.104). Multivariable analysis showed no overall subtype effect (*p* = 0.081), but triple‐negative disease had worse survival (HR 1.712, 95% CI: 1.025–2.865, *p* = 0.038).

## Discussion

### Description of intratumoural stroma

In the present study we investigated a large case series of resected BM of carcinomas and melanomas for the presence and morphological type of intratumoural stroma. Using a reproducible morphological classification system for the characterisation of different stroma patterns, we demonstrate that intratumoural stroma – with and without desmoplasia, particularly referred to as intratumoural desmoplastic stroma (IDS) – is a phenomenon specific to carcinoma BM.

Although desmoplastic and high stromal content are generally associated with more aggressive tumour behaviour across various primary carcinoma types [10, 11, 12, 13, 14, 15], intratumoural stroma in our cohort of BM showed no significant association with overall survival. Intratumoural stroma was also independent of patient age, sex, brain localisation, PDL1 expression, known driver mutations in lung cancer, or molecular subtypes in breast cancer. Nevertheless, its distribution differs among tumour types: Intratumoural stroma is most commonly observed in metastases from gastrointestinal carcinomas; however, it rarely exhibits desmoplasia in this context. In contrast, IDS is significantly more frequent in breast cancer metastases.

The term ‘stroma’ is rarely used in the context of BM. It typically refers to interactions involving the surrounding brain tissue rather than the intratumoural stroma we describe in our research. Those studies either examine the relationship between reactive glial cells and tumour cells or focus on the invasion front [16], which tends to appear rather well demarcated, often surrounded by a ‘pseudocapsule’ of reactive astrocytes, in contrast to the diffusely infiltrative growth of primary glial neoplasms [17].

However, BM has been described to show different infiltration patterns and is not always microscopically clearly demarcated with a gliotic capsule [18, 19]. Based on an autopsy series of 57 patients, Berghoff and colleagues identified for example three histopathological invasion patterns at the tumour‐brain interface: well‐demarcated growth, vascular co‐option, and diffuse infiltration. In the results part they also reported fibrous tumoural stroma in 32 out of 57 cases (56.1%), but did not specify where the stromal component was located nor did they comment on the presence or absence of desmoplastic features [20].

In a different study, Téglási and colleagues analysed infiltration patterns in a series of 40 BM, focusing on the distribution of connective tissue both peritumourally and intratumourally. Metastases exhibiting a pushing‐type infiltration displayed well‐defined tumour borders with an accumulation of collagen at the tumour‐brain interface. In contrast, BM with an irregular infiltration zone – referred to as the papillary type in their nomenclature – showed increased intratumoural vascularisation and collagen, frequently forming fibrous septa or stromal cores centrally [21].

The authors argue that pericytes generate fibrotic stroma at the invasive front. As the carcinoma continues to grow, particularly in irregularly infiltrating metastases, these stromal components are then gradually incorporated into the tumour mass and thus become intratumoural. In their study, breast cancer metastases frequently exhibited such irregular infiltrative growth patterns [21]. This observation aligns with our data, as breast carcinomas in our cohort showed a significantly higher frequency of IDS.

In a recent study, Akanda and colleagues present a proposal on the categorisation of BM using immunohistochemical analyses of stromal cells: In their pattern 1, stroma cells form a rim at the periphery of large tumour nests, a configuration the authors predominantly observed in metastases originating from kidney and liver carcinomas. Pattern 2 is characterised by stroma cells surrounding medium‐sized tumour cell clusters and is commonly seen in BM of lung, colorectal, and gastric cancers. Pattern 3 involves cells that are either intermingled with or directly encasing individual cancer cells or small tumour nests; this pattern is especially prominent in breast cancer BM. In contrast, Pattern 4 is essentially devoid of stromal fibroblasts and represents a stroma‐poor microenvironment, a feature they typically encountered in metastatic melanoma [22].

A narrow stromal rim (Akanda's pattern 1), which they described as typical for clear cell RCCs, would probably not qualify as true intratumoural stroma based on HE morphology and mostly corresponds to our stroma Group 0. Akanda's patterns 2 and 3 cannot be reliably differentiated on HE sections and are likely to overlap with our Groups 1 and 2. Consistent with our study, melanoma BM did not develop a characteristic tumour stroma (Akanda's pattern 4).

Melanomas in the brain typically grow by vascular co‐option: they align with and migrate along pre‐existing blood vessels [20, 23, 24, 25]. This supplies nutrients without building a distinct intratumoural stroma or the need for angiogenesis, consistent with our finding that melanomas rarely form classical desmoplastic stroma.

These morphological differences likely reflect fundamental tumour‐type biology. Carcinoma cells release pro‐fibrotic mediators (TGF‐β, PDGF, FGF) and chemokines (e.g., CXCL12) that recruit local mesenchymal cells and convert them into CAFs [26, 27]. These cells then deposit extracellular matrix (collagens I/III, fibronectin, tenascin‐C), forming a desmoplastic intratumoural stroma [27, 28, 29], even in the brain. Although the exact origin of CAFs in BM remains debated, single‐cell RNA sequencing of breast cancer BM has identified myofibroblastic and perivascular CAF subsets [30].

By contrast, melanoma cells, derived from the neural crest rather than epithelium, have intrinsic migratory plasticity and can metastasise without a classical epithelial‐mesenchymal transition, making them less dependent on CAF‐driven remodelling [31]. They frequently co‐opt pre‐existing brain vessels and rely on non‐fibrillar stromal interactions [32]. Notably, desmoplastic cutaneous melanomas metastasise less often [33], suggesting that dense fibrosis may restrain melanoma motility or enhance immune surveillance.

Our three‐tiered morphological classification represents a robust and practical stratification system, particularly as it can be reliably assessed on routine HE slides without requiring immunohistochemistry. We have also identified desmoplastic stroma as a distinct phenomenon for carcinoma BM, which to our knowledge has not been previously reported. It should also be noted that previous morphological studies on this subject comprised smaller case collections, not exceeding 68 cases in contrast to our study with 604 BM.

### Survival data related to tumour stroma

A high stromal content is generally associated with more aggressive tumour behaviour and poorer prognosis across various carcinoma types. The tumour stroma ratio, assessed on HE staining, has been identified as an independent prognostic factor in several malignancies, including colorectal [10], breast [14], lung [12], and hepatocellular carcinomas [13].

In addition to stromal quantity, a recent multicentre study on primary resected colorectal carcinoma demonstrated that the presence of desmoplastic stroma at the invasive front was associated with significantly worse outcomes compared to tumours with mature, non‐desmoplastic stroma [11].

In contrast, studies on ductal pancreatic carcinoma have also reported counterintuitive findings, where highly dense stroma was associated with prolonged survival. This protective effect was significant only in early‐stage, resected tumours [15].

To date, survival data specifically addressing stromal characteristics in BM are lacking in the literature. Contrary to expectations derived from primary tumour studies, no overall prognostic effect was observed between the three stroma groups in our group. However, a subsequent multiregression subgroup analysis confined to lung carcinomas revealed a significant survival advantage (*p* = 0.028) for NSCLCs exhibiting Group 1 stroma, which outperformed both carcinomas lacking intratumoural stroma (Group 0) and those with intratumoural desmoplastic stroma (IDS; Group 2).

When all BM were considered, the univariate Kaplan–Meier curves mirrored this pattern up to month 24; thereafter, however, survival for Group 1 tumours declined abruptly. Two biological explanations may account for this inflection.

First, the favourable course observed for Group 1 tumours during the initial 2‐year interval might be a chance finding, in which case the overall analysis is more accurate. Visually, after 5 years, that analysis suggests a non‐significant gradient in which Group 0 performs slightly better than Group 1, and Group 1 marginally better than Group 2. But, in the end these effects were not significant.

Alternatively, the NSCLC‐specific subgroup analysis could be closer to a biological truth, implying that the downturn in Group 1 beyond 2 years in the overall cohort is a coincidence. Under this scenario, the biological model would hypothesise that metastases with a high tumour burden and no typical intratumoural stroma (Group 0) have a significantly worse outcome than those that develop broader, but non‐desmoplastic stroma (Group 1). Those could benefit from a lower tumour burden. This advantage might then get lost once a desmoplastic stroma reaction emerges (Group 2), a pattern that has been linked to aggressive behaviour in other primary tumours as well [11, 12, 13, 14, 15].

### Additional survival data

Although we primarily focused on the morphological analysis, our large cohort nonetheless yielded several notable independent clinical observations. Median OS after neurosurgical resection was 10.3 months – about twice the 5.0 months reported for unselected SEER patients (2015–2019) [1]. This advantage reflects the favourable selection of surgical candidates – typically solitary metastasis and good performance status (KPS/ECOG) – and indicates that resection can roughly double expected survival.

Patients operated between 2015 and 2019 lived longer than those treated in 2010–2014, with significant gains for lung cancer (*p* = 0.018) and melanoma (*p* = 0.016). These improvements mirror SEER trends and likely stem from the introduction of immune‐checkpoint inhibitors and other modern systemic therapies during that period [34, 35].

Cerebellar metastases conferred a significantly worse prognosis, probably because infratentorial lesions more often cause complications such as acute hydrocephalus or haemorrhage. Carefully matched series of solitary BM show no survival difference between supratentorial and cerebellar sites [36], whereas larger unselected cohorts, including ours, consistently report poorer outcomes for infratentorial disease [37].

Finally, consistent with DS‐GPA and SEER data, triple‐negative breast cancer remained the breast‐cancer subtype with the shortest OS among brain‐metastasis patients, underscoring the lack of effective targeted treatments relative to hormone‐receptor‐ or HER2‐positive tumours [1, 2].

### Limitations

In this retrospective, descriptive study, most metastases were only partly embedded and were usually represented by one or two paraffin blocks, reflecting routine diagnostic sampling. Consequently, sampling error is unavoidable and the true frequency of intratumoural stroma – desmoplastic or otherwise – may be underestimated. Biopsies were excluded because reliable stromal evaluation is impossible in fragmented specimens. Because stromal assessment relied on archival H&E stains with routine sampling, a risk of information bias (misclassification) cannot be excluded. As a single‐centre study, generalisability is limited: surgical thresholds, pathology workflows and systemic‐therapy access vary across regions. Multicentre material would help validate our findings, and comparative studies with prospectively collected series are needed.

Our cohort was also selective: almost all lesions were solitary resected brain metastases (BM), introducing selection bias toward surgically eligible cases with adequate tissue. Because primary tumours were typically operated on in surrounding hospitals, matched primaries were rarely available, precluding detailed comparison of stromal patterns between primary and metastatic sites.

Accordingly, the present work focused on BM morphology alone. Brain parenchyma comprises neurons, glia and vascular elements but virtually no fibroblasts, whereas tumour‐associated fibroblasts, an inherently heterogeneous population, characterise many extracranial cancers [38, 39, 40, 41, 42, 43, 44]. Emerging evidence suggests that specific stromal markers may be prognostically relevant in breast‐cancer BM [44]. Comprehensive molecular, spatial and imaging‐based analyses of intracranial and matched extracranial tumours will therefore be essential to clarify the biological and clinical role of metastatic stroma.

## Conclusion

Based on a cohort of 604 resected brain metastases (BM), we established a morphology‐based, three‐tier classification system for intratumoural stroma on HE staining, comprising stroma‐poor (Group 0), fibrous non‐desmoplastic stroma (Group 1), and desmoplastic stroma (IDS, Group 2). Intratumoural stroma was only seen in carcinomas, most often in breast and pulmonary squamous‐cell metastases, and never in melanoma. A morphologically apparent desmoplastic stroma reaction appeared in approximately 25% of carcinoma metastases and was not associated with relevant clinicopathologic parameters, including survival. To our knowledge, this is the first study to document IDS as a carcinoma‐specific phenomenon in BM and to evaluate its potential prognostic relevance through survival analysis. Overall prognosis was primarily determined by clinical variables: increasing age, male sex, infratentorial location, and among breast carcinomas, the triple‐negative subtype. Findings from a subgroup analysis of non‐small‐cell lung cancer, with a potential U‐shaped relationship in which Group 1 tumours fared best, warrant validation in larger, molecularly annotated datasets and also raise the possibility that distinct stromal states may modulate tumour biology in a subtype‐specific manner.

## Author contributions statement

DB was responsible for data collection, statistical analysis and drafting the manuscript. RL, SW and AG jointly supervised the study and critically revised the manuscript, with major input by RL. PN contributed to manuscript revision and participated in the interobserver analysis as an independent evaluator. OK, KO and SW conducted neuropathological diagnosis of brain metastases in routine practice (2010–2019); the validated diagnoses underpinned this study.

## Supporting information


**Table S1.** Overview of the 42 patients with ≥1 additional brain metastasis resection(s)

## Data Availability

The datasets analyzed during the current study are available from the corresponding author on reasonable request.
