# Supplementary material for: Characterisation and impact of intratumoural stroma in melanoma and carcinoma brain metastases
Source: J Pathol Clin Res. 2025 Nov 29;12(1):e70061. doi: 10.1002/2056-4538.70061 (PMC12664527; doi:10.1002/2056-4538.70061)
Supplement: Supplementary file 1 — Table S1. Overview of the 42 patients with ≥1 additional brain metastasis resection(s) [file CJP2-12-e70061-s001.pdf]

Characterisation and impact of intratumoural stroma in melanoma and carcinoma brain metastases

D Bandke *et al*. *J Pathol Clin Res* https://doi.org/10.1002/2056-4538.70061

Supplementary Table S1

#	Sex	Primary	Stroma R1	Stroma R2	Stroma R3	Group _dif	Time R1-2	Time R2-3	Multiple BM
1	Male	Bladder	2	0		Yes	0		Synchronous
2	Female	Breast	0	2		Yes	35		Metachronous
3	Female	Breast	2	0		Yes	0		Synchronous
4	Male	Lung	0	2		Yes	12		Metachronous
5	Male	Lung	0	2		Yes	2		Metachronous
6	Male	Lung	0	2	2	Yes	6	12	Metachronous
7	Male	upper GI	0	2		Yes	3		Metachronous
8	Male	upper GI	2	0		Yes	35		Metachronous
9	Female	Bladder	0	0		No	1		Metachronous
10	Female	Breast	2	2		No	2		Metachronous
11	Female	Breast	0	0		No	0		Synchronous
12	Female	Breast	0	0		No	29		Metachronous
13	Female	Breast	0	0		No	4		Recurrence
14	Female	Breast	0	0		No	2		Recurrence
15	Female	Colon	1	1		No	8		Metachronous
16	Female	Colon	1	1		No	8		Metachronous
17	Male	Colon	1	1		No	6		Recurrence
18	Female	Kidney	0	0		No	0		Synchronous
19	Female	Lung	2	2		No	0		Synchronous
20	Male	Lung	2	2		No	0		Synchronous
21	Female	Lung	2	2		No	4		Metachronous
22	Male	Lung	2	2		No	0		Synchronous
23	Female	Lung	0	0		No	0		Synchronous
24	Male	Lung	0	0		No	0		Synchronous
25	Male	Lung	0	0		No	18		Metachronous
26	Female	Lung	2	2		No	20		Metachronous
27	Female	Lung	0	0		No	14		Metachronous
28	Male	Lung	0	0	0	No	4	7	Metachronous
29	Male	Lung	0	0		No	0		Synchronous
30	Male	Lung	0	0		No	16		Metachronous
31	Female	Lung	0	0		No	1		Metachronous
32	Male	Lung	0	0		No	28		Metachronous
33	Male	Lung	0	0		No	10		Recurrence
34	Female	Melanoma	0	0		No	14		Metachronous
35	Female	Melanoma	0	0		No	1		Recurrence
36	Male	Melanoma	0	0		No	3		Metachronous
37	Male	Melanoma	0	0		No	16		Metachronous
38	Male	Melanoma	0	0		No	20		Metachronous
39	Female	Melanoma	0	0		No	29		Metachronous

40	Male	Melanoma	0	0	0	No	1	0	Recurrence
41	Male	prostate	0	0		No	0		Synchronous
42	Female	Upper GI	1	1		No	0		Synchronous

Table S1. Overview of the 42 patients with ≥1 additional brain metastasis resection(s). For each patient, the table lists the stromal category at each resection (resection 1 = R1, resection 2 = R2, resection 3 = R3), the difference of change in stroma group, the interval between resections in months (0 = less than 1 month or even same surgery), and whether the metastases were synchronous or metachronous. In metachronous cases, available radiological information in a few cases could determine whether the lesion represented a local recurrence.
